# Ablation of Type-1 IFN Signaling in Hematopoietic Cells Confers Protection Following Traumatic Brain Injury^1^,^2^,^3^

**DOI:** 10.1523/ENEURO.0128-15.2016

**Published:** 2016-02-18

**Authors:** Ila P. Karve, Moses Zhang, Mark Habgood, Tony Frugier, Kate M. Brody, Maithili Sashindranath, C. Joakim Ek, Stephane Chappaz, Ben T. Kile, David Wright, Hong Wang, Leigh Johnston, Maria Daglas, Robert C. Ates, Robert L. Medcalf, Juliet M. Taylor, Peter J. Crack

**Affiliations:** 1 Department of Pharmacology and Therapeutics, University of Melbourne, Parkville, Victoria, Australia, 3010; 2 Australian Centre for Blood Diseases, Monash University, Melbourne, Victoria, Australia, 3004; 3 ACRF Chemical Biology Division, The Walter and Eliza Hall Institute of Medical Research, Parkville, Victoria, Australia 3052; 4 Florey Imaging, The Florey Institute of Neuroscience and Mental Health, Parkville, Victoria, Australia 3052

**Keywords:** neuroinflammation, traumatic brain injury, type-1 interferon

## Abstract

Type-1 interferons (IFNs) are pleiotropic cytokines that signal through the type-1 IFN receptor (IFNAR1). Recent literature has implicated the type-1 IFNs in disorders of the CNS. In this study, we have investigated the role of type-1 IFNs in neuroinflammation following traumatic brain injury (TBI). Using a controlled cortical impact model, TBI was induced in 8- to 10-week-old male C57BL/6J WT and IFNAR1^−/−^ mice and brains were excised to study infarct volume, inflammatory mediator release via quantitative PCR analysis and immune cell profile via immunohistochemistry. IFNAR1^−/−^ mice displayed smaller infarcts compared with WT mice after TBI. IFNAR1^−/−^ mice exhibited an altered anti-inflammatory environment compared with WT mice, with significantly reduced levels of the proinflammatory mediators TNFα, IL-1β and IL-6, an up-regulation of the anti-inflammatory mediator IL-10 and an increased activation of resident and peripheral immune cells after TBI. WT mice injected intravenously with an anti-IFNAR1 blocking monoclonal antibody (MAR1) 1 h before, 30 min after or 30 min and 2 d after TBI displayed significantly improved histological and behavioral outcome. Bone marrow chimeras demonstrated that the hematopoietic cells are a peripheral source of type-1 IFNs that drives neuroinflammation and a worsened TBI outcome. Type-1 IFN mRNA levels were confirmed to be significantly altered in human postmortem TBI brains. Together, these data demonstrate that type-1 IFN signaling is a critical pathway in the progression of neuroinflammation and presents a viable therapeutic target for the treatment of TBI.

## Significance Statement

This research article investigates the inflammatory effect of type-1 interferons (IFNs) in traumatic brain injury (TBI), in both human and mice. IFNs have been traditionally associated with peripheral inflammatory responses. However, these molecules are also present in the CNS and we believe that they play a key role in the control of neuroinflammation. Our study shows that reducing type-1 IFN signaling, either by genetic ablation or by pharmacological intervention, has a beneficial effect on the outcome after TBI. IFN signaling is required for the brain to mount an inflammatory response to the insult of TBI. This study addresses key issues in type-1 IFN signaling and is a seminal discovery of their role in TBI.

## Introduction

Traumatic brain injury (TBI) is the leading cause of death and disability in children and young adults in industrialized countries (Summers et al., 2009). There are currently no effective therapies clinically available to reduce the extent of tissue damage resulting from a TBI (McConeghy et al., 2012). TBIs are extremely heterogeneous with the extent of tissue damage highly dependent on the nature of the injury. However, a common feature of all TBIs is an initial primary lesion followed by a period of secondary tissue damage (Bramlett and Dietrich, 2004). The secondary damage seen in TBI results from complex morphological and biochemical changes in the brain that manifest following the initial impact and can last from days to weeks.

A prime player in the secondary expansion of tissue damage following TBI is neuroinflammation (Greve and Zink, 2009). Neuroinflammation is a complex phenomenon involving many different cell types and soluble factors and has been implicated as a common feature in many neuropathologies (Clausen et al., 2009; Pineau et al., 2010; Mikita et al., 2011; Wang et al., 2011). The involvement of astrocytes and resident microglia as well as peripherally invading cells adds to the complexity of TBI progression. A recent study investigating temporal neuropathological changes in humans following TBI found that neuroinflammatory events continued for years after the initial injury and contributed to ongoing neurodegeneration (Johnson et al., 2013). These ongoing neuropathological changes highlight the severity of the long-term consequences of neuroinflammation following TBI. Therefore, a better understanding of the neuroinflammatory events elicited by TBI is needed to develop more effective therapies toward limiting tissue damage and degeneration.

Type-1 interferons (IFNs) are pleiotropic cytokines that are involved in responses to viral and microbial infections and cell proliferation (Pestka, 2007). Type-1 IFNs bind to and activate the IFNα receptor (IFNAR), which is comprised of type-1 interferon receptor 1 (IFNAR1) and IFNAR2 subunits (de Weerd et al., 2007). Engagement of this receptor complex leads to signaling through the canonical JAK-STAT pathway, resulting in the up-regulation of antiviral and antiproliferative proteins, including proinflammatory cytokines and chemokines and autocrine production of type-1 IFNs (IFNα and β; Dai et al., 2011). Recent evidence indicates that injury to the CNS leads to up-regulation of type-1 IFN gene expression (Field et al., 2010). This finding suggests that type-1 IFNs may function as key mediators of the neuroinflammatory response following TBI.

In this study, we investigated the role of type-1 IFN signaling in a controlled cortical impact (CCI) model of TBI. Using mice deficient in the IFNAR1 receptor subunit (IFNAR1^−/−^), we have demonstrated that blocking type-1 IFN signaling confers neuroprotection after TBI, and significantly alters the neuroinflammatory milieu within the brain. Using a bone marrow chimeric approach, we have demonstrated that type-1 IFN signaling is involved in a deleterious role in hematopoietic cells to drive the neuroinflammatory response following TBI. Furthermore, intravenous administration of a monoclonal antibody targeting IFNAR1 (MAR1) in mice is neuroprotective, with post-TBI administration resulting in improved neurological function and decreased infarct size. Complementing this finding is data from human postmortem brains following TBI that display increased type-1 IFN levels. In total, this data supports a detrimental role for type-1 IFN signaling following TBI, and also proposes that ablation of this signaling cascade promotes the development of an anti-inflammatory, neuroprotective environment within the brain.

## Methods

### Animals

All animal procedures were performed in accordance with the Author University animal care committee’s regulations. 8- to 10-week-old male mice (23±3 g) of a C57BL/6J background were obtained from the Animal Resource Centre. IFNAR1^−/−^ mice on a C57BL/6J background were previously generated at the Institute of Reproduction and Development at Monash University and were a kind gift from Professor Paul Hertzog (Hwang et al., 1995).

### Controlled cortical impact model

Mice were anaesthetized with an intraperitoneal injection of ketamine (100 mg/kg, Parnell)/xylazine (10 mg/kg, Parnell). A sagittal scalp incision was made to expose the underlying parietal skull. A 2-mm-diameter plate of bone (centered 1.5 mm posterior to bregma and 2.5 mm lateral to the midline) was then removed using a Dremel 10.8 V drill with a 0.8 mm tip (Dremel) to expose the underlying right parietal cortex. A 1.5 mm deep impact into the exposed cortex was made at 5 m/s using the computer-controlled impactor device (LinMot-Talk 1100). Following impact, the bone plate was replaced and held in place with a small section of parafilm to cover the injury site. The skin incision was then closed with sterile silk 5.0 metric sutures (Syneture Tyco Healthcare). Mice were administered intraperitoneal buprenorphine (0.6 mg/kg, Reckitt Benckiser Healthcare) and placed on a heat mat for postsurgical recovery. Sham-operated controls underwent the same anesthesia, scalp incision, and bone plate removal, but were not injured.

### MAR1 antibody administration

WT mice were intravenously administered, in a blinded fashion, either a monoclonal antibody targeted toward IFNAR1 [anti-mouse interferon α/β receptor (IFNAR1), Leinco Technologies (MAR1), 0.5 mg] or a monoclonal antibody isotype control (IgG isotype control, Leinco Technologies, 0.5 mg) either 1 h prior to, or 30 min after TBI. In a separate cohort, WT mice were administered MAR1 or IgG both 30 min and 2 d after TBI.

### Behavioral analysis

Neurological function post-TBI was assessed using DigiGait v11.5 (Mouse Specifics) apparatus, as previously described by Sashindranath et al. (2012). Mice were run on a transparent treadmill at a speed of 15 cm/s both before injury and 3 h postinjury for 5 s. Videos of paw placement were captured in the ventral plane by the DigiGai software and analyzed by the software. All surgeries and behavioral analyses were performed in a blinded fashion. Gait measurements were calculated as postinjury–preinjury ratios of sham versus trauma mice. All gait parameters for antibody-treated mice were presented as fold-change relative to untreated TBI values.

### Preparation of serial sections for staining and infarct analysis

**Figure 1.**
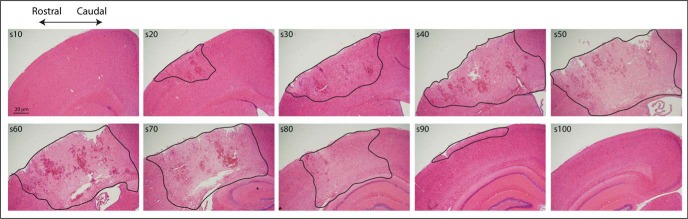
**Photomicrographs showing the full extent of the infarct in an 8- to 10-week-old C57BL6/J WT mouse.** Images are of 10-μm-thick H&E-stained sections from a mouse perfused 24 h after injury. Sections are labeled s10–s100. Damaged tissue is defined by a decrease in H&E staining intensity and an example of border demarcation is illustrated in these images. Scale bar, 20 μm.

Mice were transcardially perfused after injury (or sham surgery) with 0.1% heparinized PBS (Pfizer), followed by 4% paraformaldehyde (PFA; Scharlab S.L.). For infarct analysis, brains were collected 24 h or 7 d after TBI or sham surgery, paraffin-embedded, cut into 10 μm serial sections and mounted on glass slides (3 sections per slide). Every 10^th^ slide was stained with hematoxylin and eosin (H&E). Images of the infarct were captured using an Olympus BX50 microscope fitted with a digital camera and infarct areas were measured using ImageJ software (NIH). The volume of tissue occupied by the infarct between successive pairs of serial sections across the infarct site was calculated from the area measurements in each section and the known distance between sections (300 μm) and summed to determine total infarct volume (Fig. 1).

For immunohistochemistry, brains were dissected after perfusion at 6 and 24 h postinjury or sham surgery and placed in 4% PFA for 2 h and 10% sucrose (Univar) in PBS overnight at 4°C. Brains were then placed into optimal cutting temperature medium (Tissue Tek) and frozen in isopropanol on dry ice for a brief period and stored at −80°C until required. Brains were cut into 20 μm fresh-frozen coronal serial sections using a Microm HM 525 cryostat.

### Immunohistochemistry

Fresh frozen sections were incubated in 0.2% Triton X-100 (Sigma-Aldrich) in PBS for 20 min, and blocked for 30 min in CAS block (Invitrogen). Primary antibodies were diluted in 1% BSA (Bovogen) in PBS and slides incubated with the antibody overnight at 4°C. The primary antibodies used were as follows: CD206 (1:1000, BioScientific), Fox3 (1:200, Abcam), glial fibrillary acidic protein (GFAP; 1:1000, Cell Signaling Technologies), pSTAT3 (Ser727, 1:50, Santa Cruz Biotechnology), and Iba-1 (1:200, Wako). Slides were washed in PBS and incubated in an appropriate secondary antibody. Fluorescent secondary antibodies (AlexaFluor 594 anti-mouse and AlexaFluor 488 anti-rabbit, Invitrogen) were used at 1:1000 (diluted in PBS). Sections were mounted in Vectashield with DAPI (Vector Laboratories), and imaged under water immersion on a Leica DMI 6000B fluorescence microscope. Tiled images were imaged on a Zeiss Observer Z1 using Zen 2011 software. Quantification of GFAP and Iba-1 staining was performed using ImageJ software (NIH), as described by Baruch et al. (2014). The software generated fluorescence intensity values by tracing the region-of-interest (infarct in the ipsilateral hemisphere). Arbitrary units were defined in terms of strength of fluorescent signal. Tiled images were captured on the same day for all groups that were to be compared. In addition, fluorescence intensity analysis was done at the same time for groups that were to be compared. The final intensity values were calculated by subtracting the area of the selected region multiplied by the background fluorescence from the fluorescence intensity of the region-of-interest (ROI): fluorescence intensity (arbitrary units) = fluorescence intensity of ROI – (area of selected region × mean fluorescence of background).

### Chimera development

C57BL/6 CD45.1 or IFNAR1^−/−^ mice were irradiated (11 Gy in 2 equal doses, 2–3 h apart) to block hematopoietic cell production, as per Downes et al. (2013) with the heads of the mice shielded. Recipient animals were then intravenously injected with 1 × 10^6^ unfractionated bone marrow cells isolated from femurs of unmanipulated C57BL/6 CD45.1 or IFNAR1^−/−^ donor mice. Percentage chimerism was determined 8 weeks after bone marrow transplantation using flow cytometry to assess levels of blood leukocytes using CD45-specific monoclonal antibodies. TBI surgeries were performed 3 weeks after assessing levels of engraftment.

### Magnetic resonance imaging

Magnetic resonance imaging (MRI) scans were performed for the chimera study using a Bruker 4.7 Tesla small animal MRI scanner (Florey Institute of Neuroscience and Mental Health) to quantify the progression of tissue damage, as described by Crack et al. (2014). Mice were anesthetized with ∼3% isoflurane in a 1:1 mixture of medical-grade air and oxygen. Anesthesia was maintained throughout scanning with 0.25 to 1.5% isoflurane through a nosecone placed over the animal’s snout and respiration was continuously monitored throughout the experiment with a pressure-sensitive probe positioned under the animal’s diaphragm. Anesthetized animals were laid supine on a purpose-built small-animal holder and their heads fixed into position with ear and bite bars. A surface receiver coil was placed over the animals’ heads and the cradle was inserted into a transmitter coil fixed inside a BGA12S-HP gradient set for imaging. The MRI protocol consisted of a three-plane localizer sequence followed by multiecho T2 and diffusion-weighted sequences. The total scanning time was kept to <2 h per animal. Multiecho T2 -weighted images were acquired using a rapid acquisition, relaxation enhanced (RARE) sequence with RARE factor = 2; repetition time = 2500 ms; effective echo time (TEeff) = 10, 30, 50, 70, 90, and 110 ms; field-of-view = 1.6 Å∼ 1.6 cm^2^; matrix = 192 Å ∼192; and 16 slices with thickness = 0.5 mm. Volumetric analysis was carried out on T2-weighted images using ITK SNAP software (Yushkevich et al., 2006; www.itksnap.org).

### Analysis of human samples by quantitative PCR

All procedures were conducted in accordance with the Australian National Health & Medical Research Council’s National Statement on Ethical Conduct in Human Research (2007), the Victorian Human Tissue Act 1982, the National Code of Ethical Autopsy Practice, and the Victorian Government Policies and Practices in Relation to Postmortem. Trauma brain samples from individuals who had died following closed head injury and non-head trauma controls were obtained from the Victorian Brain Bank Network (Frugier et al., 2010, 2011,2012). Detailed patient information is outlined in Table 1. The following Taqman primers for the human tissue samples were obtained from Applied Biosciences: IFNα (ID: Hs00256882_s1), IFNβ (ID: Hs01077958_s1), IFNAR1 (ID: Hs01066118_m1), IFNAR2 (ID: Hs01022060_m1), 18S ribosomal RNA (ID: Hs99999901_s1), and UBC (Hs01871556_s1).

**Table 1:** Details of trauma and nontrauma control cases

**Details of 27 trauma and 10 control cases**						
**Case**	**Age**	**Sex**	**Cause of injury**	**PMI, h**	**Cause of death**	**Survival time**
1	51	M	Motor vehicle accident	60	Brain + multiple injuries	<17 min
2	63	M	Household accident	70	Brain injury	<17 min
3	27	M	Suicide	84	Brain + multiple injuries	<17 min
4	41	M	Suicide	96	Brain + multiple injuries	<17 min
5	57	F	Motor vehicle accident	87	Brain + multiple injuries	<17 min
6	49	M	Motor vehicle accident	107	Brain + multiple injuries	<17 min
7	45	M	Motor vehicle accident	43	Brain + multiple injuries	<17 min
8	21	M	Motor vehicle accident	100	Brain injury	<17 min
9	41.3	M	Aviation accident	114	Brain + multiple injuries	<17 min
10	57.6	F	Motor vehicle accident	97	Brain injury	<17 min
11	16.8	M	Motor vehicle accident	85	Brain + multiple injuries	<3 h
12	78.7	M	Household accident	45	Brain injury	<3 h
13	18.3	M	Motor vehicle accident	79	Brain + multiple injuries	<3 h
14	34.7	M	Motorbike accident	66	Brain + multiple injuries	<3 h
15	22.9	F	Motor vehicle accident	108	Brain + multiple injuries	<3 h
16	52.8	M	Motorbike accident	65	Brain + multiple injuries	<3 h
17	19.6	M	Suicide	33	Brain + multiple injuries	<3 h
18	59.8	M	Motor vehicle accident	71	Brain + multiple injuries	<3 h
19	46.0	M	Fall	129	Brain injury	6 h
20	56.3	M	Motor vehicle accident	65	Brain injury	8 h
21	64.6	M	Fall	61	Brain injury	8 h
22	75.9	M	Staircase fall	89	Brain injury	10 h
23	59.6	F	Motor vehicle accident	80	Brain injury	35 h
24	61.7	M	Fall	40	Brain injury	93 h
25	38.9	F	Staircase fall	101	Brain injury	122 h
26	70.9	M	Motor vehicle accident	114	Brain injury	76 h
27	73.7	M	Fall	91	Brain injury	29 h
**Controls**						
28	16	M	—	—	Suicide by hanging	—
29	48.7	M	—	50	Cardiac failure	—
30	51.6	M	—	64	Asthma	—
31	52.3	M	—	52	Cardiomyopathy	—
32	59.6	M	—	43	Pulmonary embolism	—
33	64.1	M	—	24	Ischaemic heart disease	—
34	66.9	M	—	10	Pneumonia	—
35	64.4	M	—	24	Pulmonary embolism	—
36	77.5	M	—	53	Myocardial infarction	—
**37**	60	F	—	48	Myocardial infarction	—

Cases 1–10: cases with a survival time between 0 and 17 mins; Cases 11–18: cases with a survival time between 30 min and 3 h; Cases 19–27: cases with a survival time between 6 and 261 h; Cases 28–37: control cases. All brains were obtained at autopsy. PMI, Postmortem interval (time between death and brain retrieval); M, male; F, female.

### Quantitative real-time PCR

Ipsilateral hemispheres were dissected 2, 4, and 24 h after TBI or sham surgery, and RNA was isolated using Trizol (Invitrogen). One microgram of cDNA was transcribed from RNA using a high-capacity cDNA reverse transcription kit (Applied Biosystems). Taqman and SYBR Green primers were obtained from Applied Biosciences and GeneWorks for mouse tissue samples. *C*t values were obtained for each sample, and relative transcript levels for each gene were calculated using the 2^−ΔΔ^*^C^* ^T^ method (Winer et al., 1999).

### ELISA

Ipsilateral hemispheres were dissected after TBI or sham surgery. Tissue was homogenized in Tris buffer [50 mm Tris, 150mm NaCl, 1% Triton X-100, Phospho-STOP and protease inhibitor (Roche), pH 7.4] and rotated at 4°C for 90 min. Samples were centrifuged at 2000 × *g* and supernatant was collected. To determine protein concentration of samples, a Bradford assay was performed according to the manufacturer’s instructions (Bio-Rad). Murine IL-1β, IL-6, and IL-10 ELISAs were purchased from BD Biosciences. One-hundred micrograms of protein was loaded per well in duplicate. Protein concentrations of individual samples were determined using a linear curve of muIL-1β, muIL-6, and muIL-10 standards (4–250 pg/ml).

### Statistics

Data are expressed as mean ± SEM, and analyzed using Graph Pad Prism 5.0 software. Kolmogorov–Smirnov test (with Lilliefors correction) was used to test for normality within each group. For quantitative (q) PCR, ELISA and chimera infarct analysis, a one-way or two-way ANOVA was performed where appropriate followed by Dunnett’s *post hoc* analysis and Bonferroni’s *post hoc* analysis, respectively. Infarct volume, fluorescence intensity values, and Digigait behavioral data were analyzed using an unpaired Student’s *t* test. A value of *p*<0.05 was considered significant. Statistics are summarized in Table 2.

**Table 2.** Summary of statistics from figures

Figure	Panel	Data structure#	Test type	*p* value
2	*A*^i^	Normal	Two-way ANOVA, Bonferoni *post hoc*	*p*<0.05
	*A* ^ii^	Normal	Two-way ANOVA, Bonferoni post hoc	*p*<0.001
	*A* ^iii^	Normal	Two-way ANOVA, Bonferoni *post hoc*	*p*<0.01
				
3	*B*	Normal	Students *t* test	*p*=0.0047
				
4	*A*	Normal	Two-way ANOVA, Bonferoni *post hoc*	*p*<0.001
	*C*	Normal	Two-way ANOVA, Bonferoni *post hoc*	*p*<0.05
	*D*	Normal	Two-way ANOVA, Bonferoni *post hoc*	*p*<0.05
	*E*	Normal	Two-way ANOVA, Bonferoni *post hoc*	*p*<0.05
	*F*	Normal	Two-way ANOVA, Bonferoni *post hoc*	*p*<0.01
				
5	*C*	Normal	Students *t* test	*p*=0.001
				
6	*C*	Normal	Students *t* test	*p*=0.0537
				
8	*A*	Normal	Students *t* test	*p*=0.0438
	*B*	Normal	Students *t* test	*p*=0.0001
	*C*	Normal	Students *t* test	*p*=0.0353
				
9	*A*	Normal	Two-way ANOVA, Bonferoni *post hoc*	*p*<0.001
	*B*	Normal	Two-way ANOVA, Bonferoni *post hoc*	*p*<0.05
	*C*	Normal	Two-way ANOVA, Bonferoni *post hoc*	*p*<0.05
	*D*	Normal	Two-way ANOVA, Bonferoni *post hoc*	*p*<0.05
				
10	*A*	Normal	Students *t* test	*p*=0.0277
	*B*	Normal	Students *t* test	*p*=0.0255
	*C*	Normal	Students *t* test	*p*=0.0288
	*D*	Normal	Students *t* test	*p*=0.0124
	*E*	Normal	Students *t* test	*p*=0.0156
	*F*	Normal	Students *t* test	*p*=0.0153
				
11	*C*	Normal	One-way ANOVA, Dunnett *post hoc*	*p*=0.0003
				
12	*C*	Normal	One-way ANOVA, Dunnett *post hoc*	*p*=0.0273
				
13	*C*	Normal	One-way ANOVA, Dunnett *post hoc*	*p*=0.0047
				
14	*A*	Normal	One-way ANOVA, Dunnett *post hoc*	*p*=0.0019
	*B*	Normal	One-way ANOVA, Dunnett *post hoc*	*p*=0.0001

Statistical analysis was performed using GraphPad Prism6.

#Kolmogorov–Smirnov test (with Lilliefors correction) was used to test for normality within each group.

## Results

### TBI induces type-1 IFN signaling in mice

**Figure 2.**
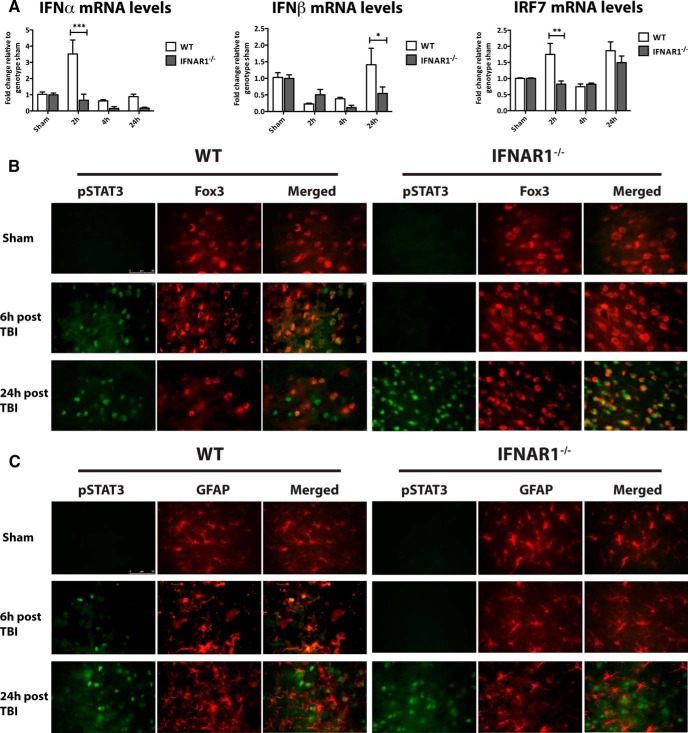
**TBI induces type-1 IFN release and downstream STAT3/IRF7 activation in an IFNAR1-dependent manner. *A*,** IFNα and IFNβ mRNA levels are elevated in ipsilateral hemispheres of WT compared with IFNAR1^−/−^ mice 2 and 24 h following TBI, respectively. Data represent mean± SEM, *n*=3 per group. *p<0.05, ***p<0.001. ***B*,** pSTAT3 immunoreactivity is observed 6 h following TBI in WT but not IFNAR1^−/−^ neuronal cells (labeled with Fox3) in the ipsilateral hemispheres compared to sham-operated mice. pSTAT3 induction is demonstrated in both WT and IFNAR1^−/−^ neurons 24h after TBI. ***C***, Six hours after TBI, pSTAT3 expression is increased in WT, but not IFNAR1^−/−^ mouse astrocytes (labeled with GFAP) in the ipsilateral hemispheres compared to sham-operated controls. pSTAT3 is expressed in both WT and IFNAR1^−/−^ astrocytes 24 h after TBI. Images are representative of *n*=3 independent experiments. Scale bar, 50 μm. **D** IRF7 mRNA levels are elevated in ipsilateral hemispheres of WT compared with IFNAR1^−/−^ mice 2 h following TBI. Data represent mean± SEM, *n*=3 per group. **p<0.01.

First, we investigated the profile of IFN transcript regulation in ipsilateral hemispheres after controlled cortical impact in mice lacking the type-1 interferon receptor, IFNAR1. IFNα expression was significantly higher in WT mice 2 h after TBI compared with IFNAR1^−/−^ mice (*p*<0.001; Fig. 2*A*). In addition, an up-regulation of IFNβ was seen 24 h after TBI in WT compared with IFNAR1^−/−^ mice (*p*<0.05; Fig. 2*A*). This confirmed release of type-1 IFNs following TBI in mice. Signal transducer and activator of transcription (STAT) 3 is a critical signal mediator in type-1 IFN signaling. STAT3 is phosphorylated or activated in this signaling cascade (Taylor et al., 2014), and here, pSTAT3 expression was used to assess the extent of type-1 IFN downstream signaling after TBI. pSTAT3 expression was assessed in neurons (stained with Fox3) and glia (stained with GFAP) in the ipsilateral hemisphere of both WT and IFNAR1^−/−^ mice (Fig. 2*B*,*C*). Fox3 was chosen as a neuronal marker because of its identification as the antigen for NeuN (neuronal nuclei; Kim et al., 2009). Six hours after TBI, pSTAT3 expression was elevated in neuronal cells in the cortex of WT mice compared with sham mice. In contrast, pSTAT3 expression could not be detected in corresponding IFNAR1^−/−^ sections. However, pSTAT3 expression was detected in both the WT and IFNAR1^−/−^ mice 24h after TBI and was colocalized with Fox3. Similarly, immunostaining identified an increase in pSTAT3 in WT mice 6 h post-TBI in glia, which was maintained 24 h post-TBI. IFNAR1^−/−^ mice demonstrated no pSTAT3 staining 6 h post-TBI, but showed pSTAT3 immunoreactivity 24 h post-TBI, some of which colocalized with glia. This suggests STAT3 may be activated through alternate pathways at later time points in the IFNAR1^−/−^ after TBI. Additionally, we investigated interferon regulatory factor 7 (IRF7) mRNA levels following TBI. IRF7 is a key protein involved in type-1 IFN induction through various pathways, one of them being the type-1 IFN pathway itself (Marié et al., 1998; Sato et al., 1998; Honda et al., 2005). We identified an increase in IRF7 levels 2h post-TBI in WT, but not IFNAR1^−/−^ mice (*p*<0.01; Fig. 2*A*). This further confirmed activation of downstream mediators of the type-1 IFN pathway in an IFNAR1-dependent manner.

### Mice lacking the IFNAR1 subunit have smaller infarct volumes after TBI

**Figure 3.**
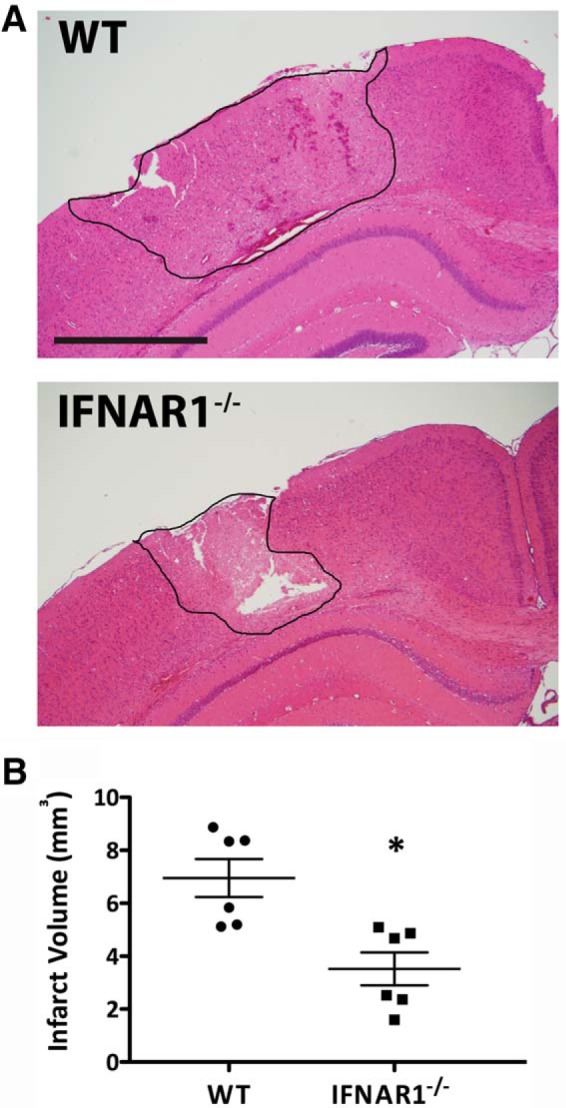
**Absence of IFNAR1 contributes to a smaller infarct volume in mice 24 h after TBI. A** Representative 10-µm-thick H&E-stained coronal brain section from a WT and IFNAR1^−/−^ mouse given TBI. **B** IFNAR1^−/−^ mice have significantly reduced infarct volumes compared with WT mice 24 h after TBI. Data represent mean ± SEM. *p<0.0; *n*=6 animals per group. Scale bar, 2 mm.

The role of IFNAR in neuroprotection after TBI was of particular interest given we had established the involvement of type-1 IFN signaling in TBI in mice. To investigate this, WT and IFNAR1^−/−^ mice were given TBI, and infarct volumes were measured in coronal H&E-stained brain sections taken 24 h post-TBI. This experimental focal model of TBI produced a lesion confined to the cortical region of the ipsilateral hemisphere (Fig. 3*A*). Twenty-four hours after TBI, IFNAR1^−/−^ mice had significantly smaller infarct volumes compared to their WT counterparts (3.52 mm^3^ compared with 6.96 mm^3^, respectively; *p*=0.0047, *n*=6; Fig. 3*B*).

### IFNAR1^−/−^ mice display lower proinflammatory, and higher anti-inflammatory cytokine levels compared with WT mice after TBI

**Figure 4.**
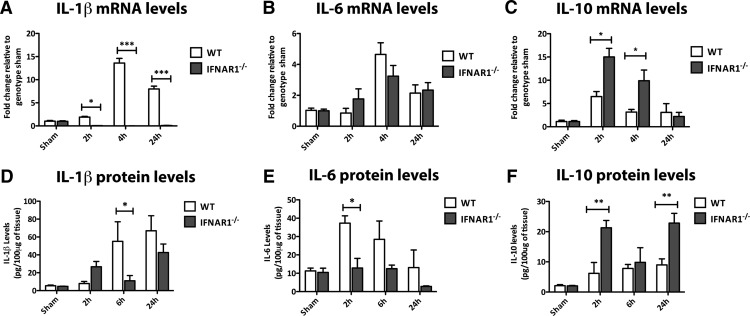
**IFNAR1^−/−^ mice display lower levels of proinflammatory cytokines and higher levels of the anti-inflammatory cytokine IL-10 in ipsilateral hemispheres compared to WT mice after TBI. *A*,** IL-1β mRNA levels are elevated at 2, 4, and 24 h after TBI in WT but not IFNAR1^−/−^ mice. ***B*,** IL-6 mRNA levels are elevated 4 h after TBI in both WT and IFNAR1^−/−^ mice. ***C*,** IL-10 mRNA levels are elevated 2 and 4 h after TBI in IFNAR1^−/−^ compared with WT mice. ***D*,** WT mice display elevated IL-1β protein compared with IFNAR1^−/−^ mice 6 h after TBI. ***E*,** IL-6 protein levels are elevated in WT compared with IFNAR1^−/−^ mice 2 h after TBI. ***F*,** IFNAR1^−/−^ mice display higher levels of IL-10 protein compared to WT mice 2 and 24 h after TBI. Data represent mean ± SEM, *n*=3 per group. *p<0.05, **p<0.01, ***p<0.001.

To investigate the mechanism behind the neuroprotection seen in IFNAR1^−/−^ mice, we investigated the expression profile of the proinflammatory genes IL-1β and IL-6 and the anti-inflammatory cytokine IL-10 in the ipsilateral hemispheres following TBI in both WT and IFNAR1^−/−^ mice. mRNA levels of IL-1β were significantly elevated in WT, compared to IFNAR1^−/−^ mice at 2, 4, and 24 h after TBI (*p*<0.05 and *p*<0.001, *n*=3; Fig. 4*A*). IL-6 was elevated in the ipsilateral hemisphere WT mice 4h after TBI compared with controls, but this up-regulation was not significantly different compared with the IFNAR1^−/−^ mice at 4 h (Fig. 4*B*). IFNAR1^−/−^ mice demonstrated increased IL-10 mRNA levels compared with WT mice both 2 and 4 h after TBI (*p*<0.05; Fig. 4*C*). To validate these data, we performed ELISAs to detect protein levels of the same cytokines. IL-1β and IL-6 protein levels were elevated in WT, compared to IFNAR1^−/−^ mice at 6 h (IL-1β) and 2 h (IL-6; *p*<0.05; Fig. 4*D*,*E*). IL-10 protein levels were elevated in IFNAR1^−/−^ compared with WT mice both 2 and 24 h post-TBI (*p*<0.01; Fig. 4*F*). These data imply signaling through IFNAR1 mediates the up-regulation of proinflammatory genes post-TBI. In addition, absence of IFNAR1 results in an increase in the anti-inflammatory cytokine, IL-10. This strongly suggests the preference for a heightened anti-inflammatory and suppressed proinflammatory response in these IFNAR1^−/−^ mice.

### IFNAR1^−/−^ mice display an increase in GFAP and Iba-1 staining compared with WT mice

**Figure 5.**
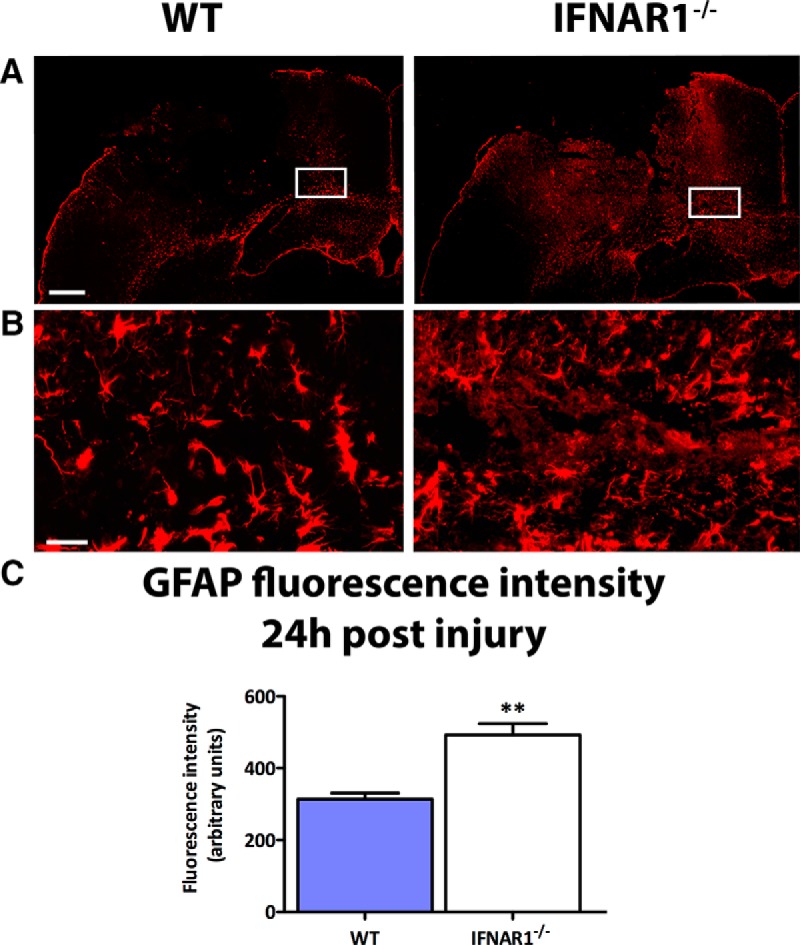
**IFNAR1^−/−^ mice exhibit increased GFAP staining compared with WT mice after TBI. *A*,** Representative image of GFAP staining in the ipsilateral hemisphere of WT and IFNAR1^−/−^ mice 24 h after TBI. Scale bar, 200 μm. ***B*,** High-resolution image of GFAP staining in the ipsilateral hemisphere of WT and IFNAR1^−/−^ mice 24 h after TBI. Image region is outlined in the white box in ***A***. Scale bar, 50 μm. ***C*,** Quantification of GFAP staining in TBI mice, using fluorescence intensity values to quantify GFAP levels. Data represent mean ± SEM, *n*=5 per group. **p<0.01.

**Figure 6.**
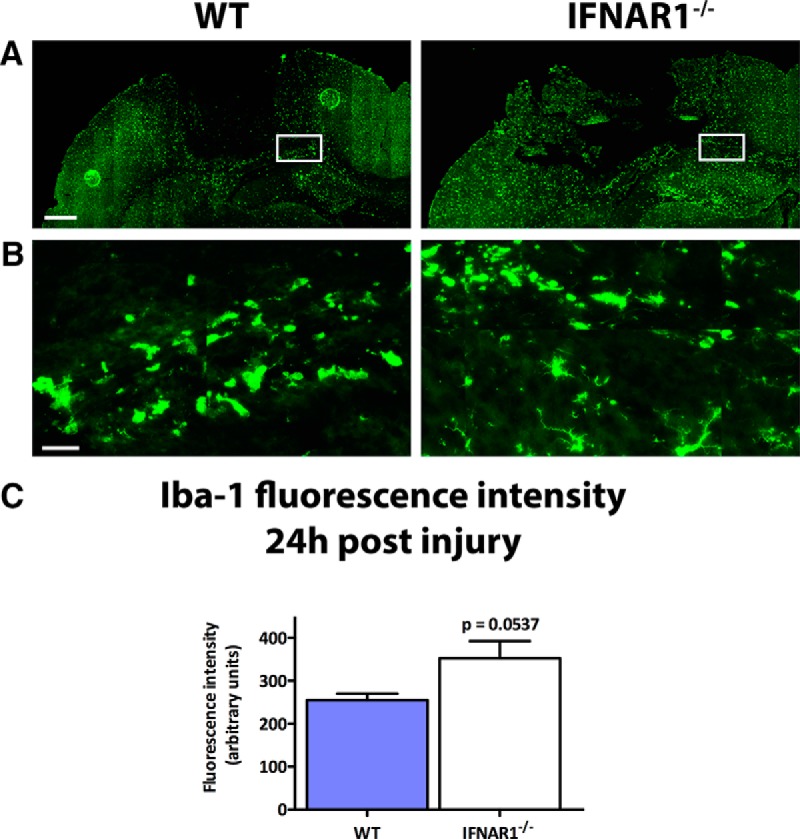
**Iba-1 levels increase after TBI, and are influenced by type-1 IFN signaling. *A*,** Representative image of Iba-1 staining in the ipsilateral hemisphere of WT and IFNAR1^−/−^ mice 24 h after TBI. Scale bar, 200 μm. ***B*,** High-resolution image of Iba-1 staining in the ipsilateral hemisphere of WT and IFNAR1^−/−^ mice 24 h after TBI. Image region is outlined in the white box in ***A***. Scale bar, 50 μm. ***C*,** Quantification of Iba-1 staining in TBI mice using fluorescence intensity values to quantify Iba-1 levels. Data represent mean ± SEM, *n*=5 per group. *p*=0.0537.

**Figure 7.**
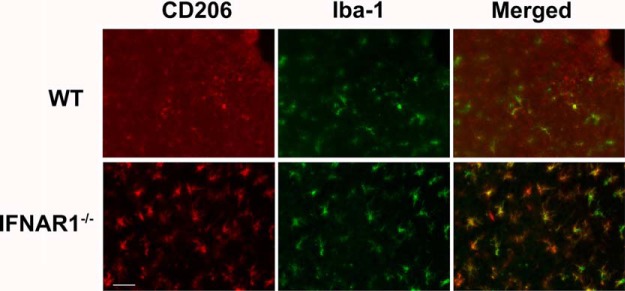
**IFNAR1^−/−^ microglia exhibit increased CD206 staining compared with WT mice after TBI.** CD206 immunoreactivity is observed following TBI in IFNAR1^−/−^ mice at a greater level compared with WT mice 24 h after TBI. CD206 immunoreactivity is colabeled with the microglial marker Iba-1.

Astrogliosis is a common feature of neuroinflammatory pathologies, and is characterized by an up-regulation in GFAP expression in astrocytes (Zamanian et al., 2012). This response may be either protective or deleterious in various pathologies (Terai et al., 2001; Paintlia et al., 2013). In WT and IFNAR1^−/−^ mice subjected to TBI, an increase in GFAP staining was seen compared to sham-operated controls indicating a neuroinflammatory response elicited by the TBI (data not shown). Interestingly, IFNAR1^−/−^ mice displayed increased GFAP staining (Fig. 5*A*) and expression compared to WT mice 24h after TBI (492.2 compared to 313.9 arbitrary fluorescence intensity units, respectively, *p*=0.001, *n*=5; Fig. 5*C*). In addition, we performed immunohistochemistry to detect activated microglia and peripherally invading macrophages. Ionized calcium-binding adapter molecule 1 (Iba-1) is expressed in both microglia and macrophages (Fukuda et al., 1996), and its expression is up-regulated by the activation of these cell types (microgliosis). Baseline Iba-1 levels were similar in WT and IFNAR1^−/−^ shams (data not shown), and were increased in both WT and IFNAR1^−/−^ mice following TBI compared with shams (Fig. 6*A*). Additionally, we observed a trend for increased Iba-1 immunoreactivity in IFNAR1^−/−^ mice compared with WT mice 24 h after TBI (352.2 compared to 254.8 arbitrary fluorescence intensity units, respectively, *p*=0.053; Fig. 6*C*). In the IFNAR1^−/−^ mice we were expecting to see decreased immunoreactivity of both GFAP and Iba-1, however contrary to our hypothesis, we identified increased immunoreactivity of both astrocytes (GFAP) and microglia/macrophages (Iba-1) in IFNAR1^−/−^ mice, suggestive of increased astrogliosis and microgliosis. However, in conjunction with this increased GFAP and Iba-1 cellular response, we identified a decreased proinflammatory and increased anti-inflammatory response (Fig. 3). The M2 marker, CD206 was shown to have staining in the IFNAR1^−/−^ brain after TBI compared with the WT, which is suggestive of an altered microglial phenotype (Fig. 7). Collectively, these data support the presence of a dominant anti-inflammatory and potentially protective environment in the IFNAR1^−/−^ mice following TBI.

### MAR1, a monoclonal antibody targeted to IFNAR1, is effective at reducing infarct volume when administered before and after TBI in mice

**Figure 8.**
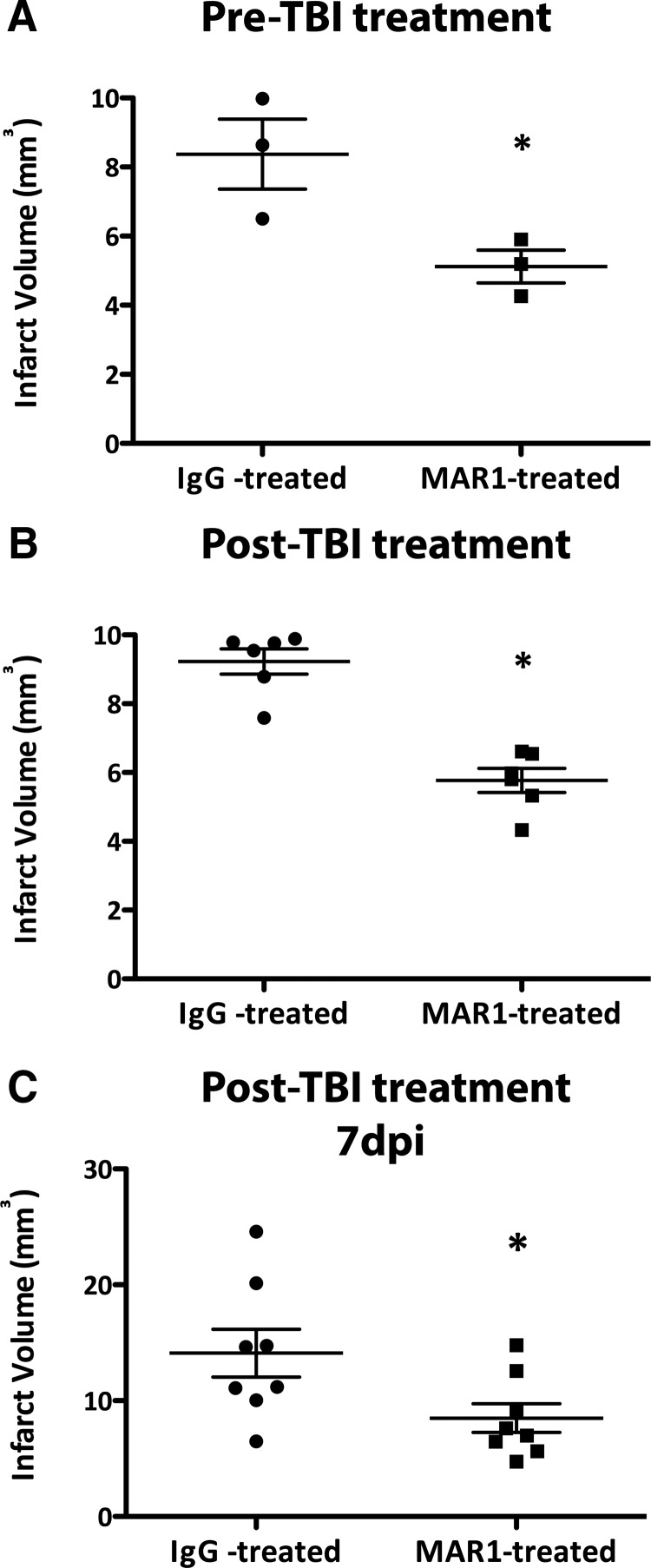
**Pre- and post-treatment with MAR1 decreases infarct volume in WT mice given TBI both 24 h and 7 d after TBI. *A*,** WT mice were treated 1 h before surgery with MAR1 (0.5 mg) or an IgG isotype control (0.5 mg); infarct was calculated 24 h after TBI. Data represent mean ± SEM; *p<0.05, *n*=3 animals per group. ***B*,** WT mice were treated 30 min after TBI with MAR1 or an IgG isotype control; infarct was calculated 24 h after TBI. Data represent mean ± SEM; *p<0.05, *n*=6 animals per group. ***C*,** WT mice were treated 30 min and 2 d after TBI with MAR1 or an IgG isotype control; infarct was calculated 7 d after TBI. Data represent mean ± SEM; *p<0.05, *n*=8 animals per group.

The neuroprotective effects of knocking out IFNAR1 were confirmed by administering an antibody to IFNAR1 (MAR1) to block the receptor either before or after injury. Pre-treatment with MAR1 1 h before injury resulted in significantly smaller infarct volumes compared to animals pretreated with an IgG isotype control (5.12 mm^3^ compared to 8.37 mm^3^, *p*<0.0438, *n*=3; Fig. 8*A*). Post-treatment with MAR1 30 min after injury also resulted in smaller infarct volumes compared to IgG isotype control-treated mice (5.77 mm^3^ compared to 9.23 mm^3^, *p*=0.0001, *n*=6; Fig. 8*B*). Additionally, when mice were treated with IgG or MAR1 both 30 min and 2 d after TBI, MAR1-treated mice had smaller infarcts compared to IgG-treated mice 7 d after TBI (8.50 mm^3^ compared to 14.11 mm^3^, *p*=0.035, *n*=8; Fig. 8*C*). These results indicate that blocking type-1 IFN signaling is neuroprotective both over a short and long time course after TBI, highlighting the therapeutic potential of MAR1 for TBI treatment.

### MAR1 administration results in decreased IFN levels and a dampened proinflammatory response

**Figure 9.**
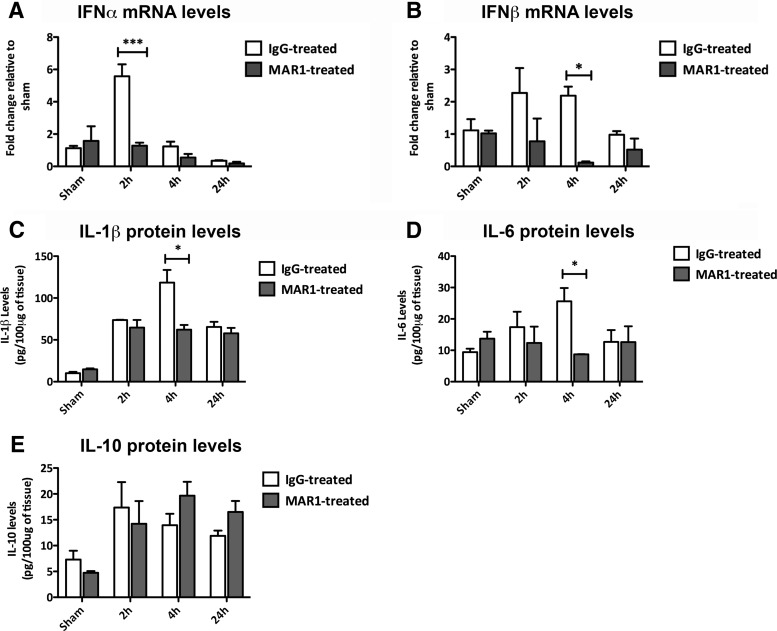
**MAR1-treated mice display reduced type-1 IFN and proinflammatory cytokine secretion following TBI. *A*,** IFNα mRNA levels are elevated in IgG-treated mice, compared to MAR1-treated mice 2 h after TBI. ***B*,** IFNβ mRNA levels are elevated in IgG-treated mice compared with MAR1-treated mice 4 h after TBI. ***C*,** IL-1β protein levels are elevated in IgG-treated mice, compared to MAR1-treated mice 4 h after TBI. ***D*,** IL-6 protein levels are elevated in IgG-treated mice compared with MAR1-treated mice 4 h after TBI. ***E***, IL-10 protein levels are unchanged by MAR1 treatment after TBI. Data represent mean ± SEM, *n*=3 per group. *p<0.05, ***p<0.001.

To investigate the mechanism of MAR1-elicited protection, we performed qPCR and ELISAs to detect levels of IFN and the proinflammatory cytokines IL-1β and IL-6. Again, we investigated changes in ipsilateral hemispheres of mice after TBI. IFNα mRNA levels were increased in IgG-treated mice compared with MAR1-treated mice 2 h post-TBI (*p*<0.001; Fig. 9*A*). IFNβ mRNA levels were increased in IgG-treated mice compared with MAR1-treated mice 4h after TBI (*p*<0.05; Fig. 9*B*). Levels of IL-1β and IL-6 were higher in IgG-treated mice compared to MAR1-treated mice 4 h post-TBI, as measured by ELISA (*p*<0.05; Fig. 9*C*,*D*), interestingly IL-10 levels were found to be unchanged by MAR1 treatment (Fig. 9*E*). Similar to genetic ablation of IFNAR1, MAR1 administration suppressed the signaling of type-1 IFNs and proinflammatory cytokines following TBI.

### Post-TBI administration of MAR1 significantly improves neurological function in injured mice

**Figure 10.**
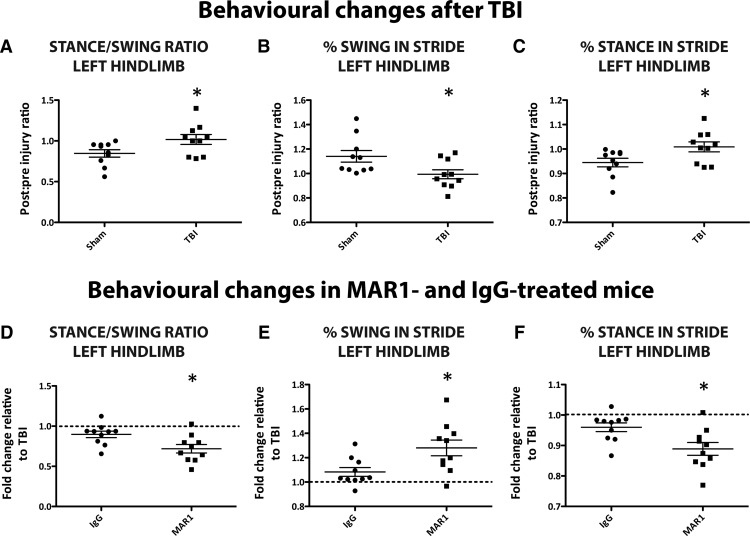
**MAR1 post-treatment significantly improves behavioral outcome after TBI.** Three hours after surgery, TBI mice show significant behavioral impairment compared to sham mice in left hindlimb parameters such as stance–swing ratio, percentage swing in stride, and percentage stance in stride (***A***–***C***). Data are presented as values of postinjury–preinjury ratios. Post-TBI administration of MAR1 significantly improves behavioral outcome compared to IgG-treated mice in these parameters (***D***–***F***). MAR1 and IgG-treated values are presented as fold-change to TBI. Data represent mean± SEM; *p<0.05, *n*=10 animals per group.

To identify changes in neurological function in mice following TBI, the Digigait™ system and software were used. Injured mice displayed impaired locomotor function in their left hindlimb (contralateral limb to injury) compared to sham operated control mice in parameters, such as stance–swing ratio, percentage swing in stride, and percentage stance in stride 3 h after TBI. Sham and TBI comparisons are presented as postinjury–preinjury ratios in Figure 10*A*–*C*. Changes in these gait indices in the MAR1 antibody and IgG isotype-treated mice are presented as fold-change relative to untreated WT TBI mice. We found that administration of MAR1 30 min post-TBI significantly improved locomotor function after TBI for the left hindlimb in the parameters of stance–swing ratio (Fig. 10*D*; *p*=0.0124, *n*=10), percentage swing in stride (Fig. 10*E*; *p*=0.0156, *n*=10), and percentage stance in stride (Fig. 10*F*; *p*=0.0124, *n*=10). These results indicate that MAR1 is an effective treatment that improves neurological function and behavioral outcome after TBI.

### Blocking type-1 IFN signaling in the hematopoietic cell compartment is protective following TBI

**Figure 11.**
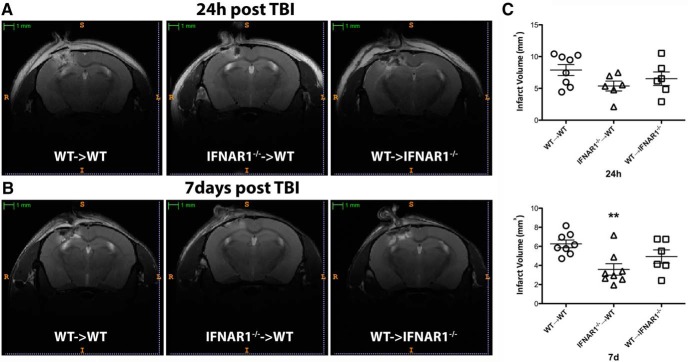
**Blocking type-1 IFN signaling in hematopoietic cells engenders protection following TBI. *A*,** MRI T2 images from bone marrow chimeric mice showing TBI lesion 24 h after injury. WT&cenveo_unknown_entity_wingdings_F0E0;WT mice represent C57BL/6 CD45.1 mice irradiated to abolish hematopoietic cells and reconstituted with BL/6 bone marrow. IFNAR1^−/−^&cenveo_unknown_entity_wingdings_F0E0;WT mice represent irradiated C57BL/6 CD45.1 mice reconstituted with IFNAR1^−/−^ bone marrow. WT&cenveo_unknown_entity_wingdings_F0E0;IFNAR1^−/−^ mice represent irradiated IFNAR1^−/−^ mice reconstituted with C57BL/6 CD45.1 bone marrow. Scale bar, 1 mm. ***B*,** MRI T2 images from chimeric mice showing TBI lesion 7 d after injury. ***C*,** IFNAR1^−/−^&cenveo_unknown_entity_wingdings_F0E0;WT mice have significantly reduced infarct volumes 7 d after injury. Data represent mean± SEM; **p<0.01, *n*=6–8 animals per group.

To dissect the cellular mechanisms behind IFNAR1-mediated neuroinflammation, we generated bone marrow chimeras of WT C57BL/6 CD45.1 and IFNAR1^−/−^ mice. We performed TBI on three groups of mice: mice, which had IFNAR1 deleted, except in hematopoietic cells (WT&cenveo_unknown_entity_wingdings_F0E0;IFNAR1^−/−^), mice, which had IFNAR1 deleted only in hematopoietic cells (IFNAR1^−/−^&cenveo_unknown_entity_wingdings_F0E0;WT), and mice without IFNAR1 deletion (WT&cenveo_unknown_entity_wingdings_F0E0;WT). We assessed successful engraftment of donor cells using flow cytometry. All groups of mice demonstrated >95% engraftment in CD19-positive blood B cells and ∼80% engraftment of CD19-negative T cells (data not shown). Engraftment levels were measured 8 weeks post-transplantation. The level of engraftment in T cells reflects the fact that the T cells were largely resistant to the irradiation. WT radiation naïve mice displayed similar infarct volumes to WT mice reconstituted with WT bone marrow (data not shown). T2-weighted MRI demonstrated that the IFNAR1^−/−^&cenveo_unknown_entity_wingdings_F0E0;WT group had significantly lower infarct volumes 7d following injury, and displayed a trend for a reduced infarct volume 24 h following injury (Fig. 11*A*,*C*). In comparison there was no significant change in infarct volume in the WT&cenveo_unknown_entity_wingdings_F0E0;IFNAR1^−/−^ group (Fig. 11*B*,*C*). This finding reveals a critical role for type-1 IFN signaling in driving neuroinflammation in peripheral hematopoietic cells following TBI.

### Type-1 interferon signaling in the hematopoietic compartment influences astrogliosis and microgliosis

**Figure 12.**
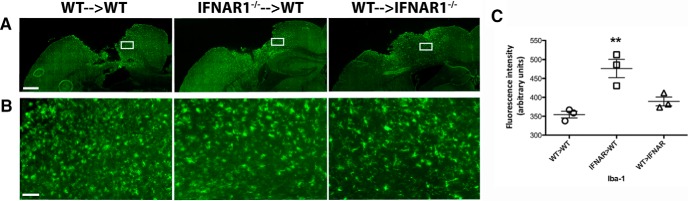
**GFAP immunoreactivity is significantly elevated in IFNAR1^−/−^**&cenveo_unknown_entity_wingdings_F0E0;**WT mice 7 d after TBI. *A*,** Representative tiled images using GFAP immunohistochemistry in chimera groups 7 d after TBI. Scale bar, 200 μm. ***B***, High-resolution image of GFAP staining in the ipsilateral hemisphere of all chimeras 7 d after TBI. Image region is outlined in the white box in ***A***. Scale bar, 50 μm. ***C*,** Quantification of GFAP staining in TBI mice, using fluorescence intensity values to quantify GFAP levels. Data represent mean ± SEM; *n*=3 per group, *p=0.0273.

**Figure 13.**
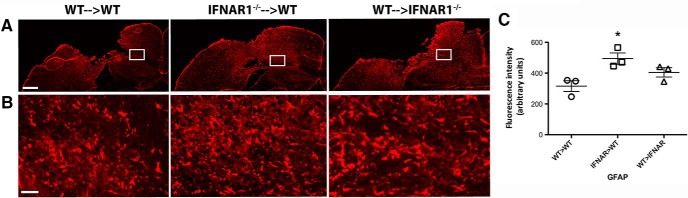
**Iba-1 immunoreactivity is significantly elevated in IFNAR1^−/−^**&cenveo_unknown_entity_wingdings_F0E0;**WT mice 7 d after TBI. *A*,** Representative tiled images using Iba-1 immunohistochemistry in chimera groups 7 d after TBI. Scale bar, 200 μm. ***B***, High-resolution image of Iba-1 staining in the ipsilateral hemisphere of all chimeras 7 d after TBI. Image region is outlined in the white box in ***A***. Scale bar, 50 μm. ***C*,** Quantification of Iba-1 staining in TBI mice using fluorescence intensity values to quantify Iba-1 levels. Data represent mean ± SEM; *n*=3 per group, **p=0.0047.

An increase in GFAP and Iba-1 staining was observed in TBI mice compared with sham-operated chimeras (sham tiled images not shown). GFAP immunoreactivity was unchanged between all chimera groups 24 h after injury when quantified as fluorescence intensity (data not shown). Seven days postinjury, IFNAR1^−/−^&cenveo_unknown_entity_wingdings_F0E0;WT mice displayed elevated GFAP levels compared with WT&cenveo_unknown_entity_wingdings_F0E0;WT and WT&cenveo_unknown_entity_wingdings_F0E0;IFNAR1^−/−^ mice (494.4 ± 36.5 arbitrary fluorescence units (IFNAR1^−/−^&cenveo_unknown_entity_wingdings_F0E0;WT), 315.9 ± 34.6 arbitrary fluorescence units (WT&cenveo_unknown_entity_wingdings_F0E0;WT), and 404.9 ± 30.0 arbitrary fluorescence units (WT&cenveo_unknown_entity_wingdings_F0E0;IFNAR1^−/−^); *p*=0.0273; Fig. 12]. Similarly, no identifiable differences were observed with Iba-1 immunohistochemistry in chimera groups at 24 h (data not shown). Again, 7 d postinjury, IFNAR1^−/−^&cenveo_unknown_entity_wingdings_F0E0;WT mice displayed significantly elevated Iba-1 levels compared to the other two groups [476.4 ± 24.2 arbitrary fluorescence units (IFNAR1^−/−^&cenveo_unknown_entity_wingdings_F0E0;WT), 354.5 ± 8.7 arbitrary fluorescence units (WT&cenveo_unknown_entity_wingdings_F0E0;WT), and 389.3 ± 11.45 arbitrary fluorescence units (WT&cenveo_unknown_entity_wingdings_F0E0;IFNAR1^−/−^); *p*<0.0047;Fig. 13]. Elevated GFAP and Iba-1 levels in IFNAR1^−/−^&cenveo_unknown_entity_wingdings_F0E0;WT chimeras could indicate an increased accumulation of reactive astrocytes and microglia/macrophages in these mice, especially in the days following injury.

### Type-1 interferons are involved in human TBI pathology

**Figure 14.**
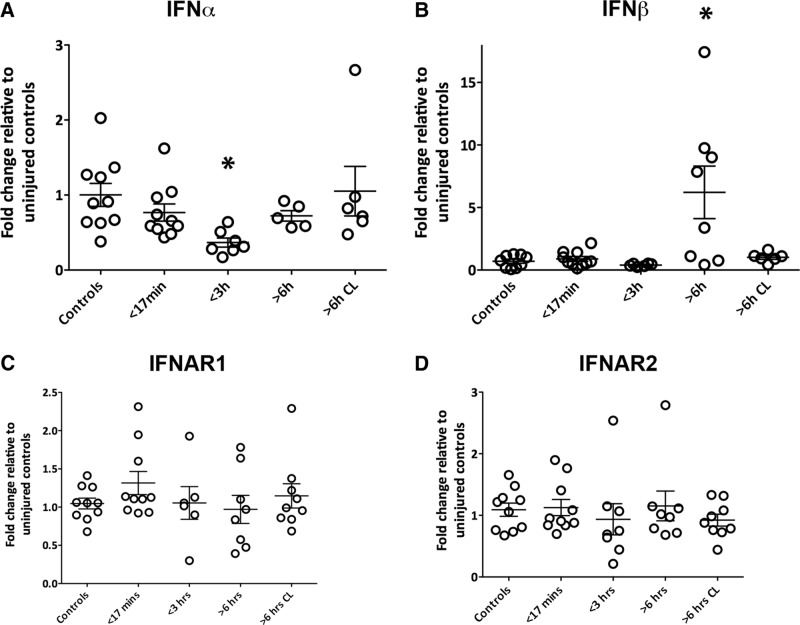
**Type-1 IFN transcript levels are altered in humans after TBI, whereas receptor levels are unchanged. *A*,** IFNα mRNA levels are decreased compared with control in patients who died <3 h after TBI. ***B*,** IFNβ mRNA levels are elevated compared to control in patients who died >6 h after TBI. **C** IFNAR1 and IFNAR2 mRNA levels are unchanged in postmortem brains. Samples were taken from the ipsilateral hemisphere of these patients. Samples from the contralateral hemisphere of patients who died >6 h after TBI are represented in the last bar (>6 h CL). Data represent mean ± SEM; *n*=5–11 patients per group, *p<0.05.

To investigate the contribution of type-1 IFN signaling in humans following TBI, we assessed type-1 IFN mRNA levels in postmortem brains with qPCR (Fig. 14). Details of human postmortem tissue samples can be found in Table 1. A decrease in IFNα mRNA levels was identified in subjects that had died 3 h after TBI (*p*=0.0019; Fig. 14*A*). In contrast, IFNβ mRNA levels were significantly increased in subjects that had died 6 h after TBI compared with controls (*p*=0.0001; Fig. 14*B*). Levels of the receptor subunits IFNAR1 and IFNAR2 remained unchanged in injured brains compared with controls, indicating the potential for intact ligand-receptor interaction (Fig. 14*C*). Type-1 IFNs are therefore implicated in humans after TBI, demonstrating the relevance of studying this system in neuroinflammation following TBI.

## Discussion

The current data show that ablation of IFN signaling through genetic deletion of IFNAR1 or pharmacological blockade of the receptor leads to pronounced protection after TBI. Previous studies have documented the roles of type-1 IFNs in responses to viral and tumor-associated pathologies (Hwang et al., 1995; Henry et al., 2007) but this is the first study to implicate type-1 IFN signaling in *in vivo* acute neural injury. Type-1 IFNs are known to be released in response to cellular stress via toll-like receptor (TLR) pathways (Field et al., 2010), contributing to further damage and neurodegeneration (Taylor et al., 2014). The role of type-1 IFNs in the CNS is an emerging field of study, with recent evidence suggesting that the type-1 IFN response contributes to the pathology seen in acute and chronic neuropathologies (Khorooshi and Owens, 2010; Wang et al., 2011; Taylor et al., 2014).

Our study highlights the involvement of type-1 IFN signaling in both mice and humans following TBI. We reported IFNAR1-dependent increases in IFNα and IFNβ after TBI in mice. In addition to primary type-1 IFN induction, it is known that type-1 IFNs are also produced through IFNAR signaling as a positive-feedback mechanism (Gough et al., 2010). It is also possible that IFNAR drives the secondary production of these IFNs leading to the exacerbation of inflammation in humans. Interestingly, IFNα was elevated early in mice, contrasting to the down-regulation in IFNα mRNA in human TBI patients. In addition to this, IFNβ was elevated in humans to a greater extent than that seen in mice. The disparity between the human and murine results may be explained as the initiator of the neuroinflammatory cascade may differ; being IFNβ in humans and IFNα in mice. The production of IFNs is under tight regulation by IFN-producing pathways. It has been established that murine type-1 IFN release is controlled largely by the transcription factor IRF7 (Honda et al., 2005). A recent study in human blood monocytes demonstrated that IFNβ production was under joint control of the transcription factors IRF3 and IRF8 (Li et al., 2011). Although these studies were conducted in models of infection, they do suggest that the regulation of type-1 IFN induction differs between mice and humans; hence, the production of these cytokines will be influenced largely by which IRFs are dominant following infection or injury.

Downstream activation of type-1 IFN signaling was further confirmed after TBI with pSTAT3 immunohistochemistry and IRF7 induction. STAT3, a transcription factor, has broad roles in cell cycle regulation, and can be activated via IFN signaling pathways (Taylor et al., 2014). Recently, STAT3 phosphorylation was identified in astrocytes in a rat fluid-percussion injury model of TBI where it was proposed that activation of STAT3 could contribute to inflammation or be neuroprotective depending on cell type (Oliva et al., 2012). In addition, STAT3 was found to be phosphorylated in an IFNAR1-dependent manner in a model of Alzheimer’s disease, identifying STAT3 as a crucial downstream effector of type-1 IFN signaling (Taylor et al., 2014). In our CCI model, it was found that STAT3 was phosphorylated in GFAP-positive astrocytes and Fox3-positive neurons 6 h following injury in an IFNAR1-dependent manner. This activation was absent in IFNAR1^−/−^ brains. Our findings support a role for STAT3 as a critical downstream mediator of type-1 IFN signaling following CNS injury. In addition, IRF7 was induced 2 h following TBI in WT, but not IFNAR1^−/−^ mice. IRF7 is implicated in type-1 IFN production and signaling, and it has been shown that absence of IRF7 impairs IFNα and β production (Honda et al., 2005). In a study of hippocampal sterile injury, type-1 IFN signaling pathways were activated via IRF7, leading to the release of downstream inflammatory mediators (Khorooshi and Owens, 2010). Collectively, our results point to an engagement and activation of type-1 IFN signaling pathways following TBI.

Downstream of type-1 IFN pathway activation, we investigated the release of proinflammatory mediators IL-1β and IL-6. Type-1 IFN signaling influenced the release of these mediators, with diminished levels of IL-1β mRNA and both IL-1β and IL-6 protein in the IFNAR1^−/−^ mice. The neutralization of proinflammatory cytokines has often been associated with beneficial outcomes post-TBI (Clausen et al., 2009). Additionally, studies investigating therapeutics targeting inflammation post-TBI often report decreases in proinflammatory cytokine levels (Truettner et al., 2005; Lloyd et al., 2008). Together, this evidence proposes that suppression of the proinflammatory response in the IFNAR1^−/−^ mice after TBI could explain a potential mechanism as to why these mice exhibit decreased lesion volumes.

Another crucial hallmark of the neuroinflammatory cascade in TBI is the activation of resident astrocytes and microglia, and infiltration of peripheral immune cells (D'Mello et al., 2009; Pineau et al., 2010; Zamanian et al., 2012; Wang et al., 2013). Astrocytes are crucial in regulating responses to infection and neural injury, and respond to such challenges by becoming “reactive” and up-regulating expression of GFAP, in a process termed astrogliosis (Zamanian et al., 2012). Astrogliosis has been defined in the context of both neurodegeneration and neuroprotection in the CNS, and reactive astrocytes are known to produce mediators, such as proinflammatory and anti-inflammatory cytokines and growth factors, to elicit their effects onto the surrounding environment (Myer et al., 2006; Zamanian et al., 2012). Similarly, microglial cells can also undergo reactive gliosis under conditions of cellular stress or injury (Cao et al., 2012). In contrast to our results reporting decreased proinflammatory cytokines, we observed increased GFAP and Iba-1 staining in the IFNAR1^−/−^ mouse after TBI, indicative of increased astrogliosis and microgliosis. Interestingly however, this increased gliosis in IFNAR1^−/−^ mice was observed concomitantly with diminished levels of proinflammatory mediators, and elevated levels of anti-inflammatory IL-10, suggestive of an “M2-like” or reparative glial response to injury.

The Iba-1 antibody is known to detect both microglia and peripherally invading macrophages; therefore, it is reasonable to expect the detection of both cell types in our TBI model. A recent study conducted in a mouse compression injury model of TBI demonstrated that an acute inflammatory response coordinated primarily by microglia, macrophages and neutrophils is neuroprotective and limits cell death within the meninges and deeper brain tissue (Roth et al., 2014). The increase in microglia and macrophages found in the IFNAR1^−/−^ mice following TBI may be eliciting a similar neuroprotective function. Similar to the astrocytes in the IFNAR1^−/−^ mice, activated microglia/macrophages in the absence of IFNAR1 may lack the ability to produce proinflammatory cytokines. This may explain why IFNAR1^−/−^ mice have a dramatically reduced cytokine load after TBI. These results highlight the possibility of distinctive differences in the neuroinflammatory milieu between WT and IFNAR1^−/−^ mice after TBI.

Our results suggest that the IFNAR1^−/−^ mice demonstrate a greater M2-polarized environment compared to WT mice after TBI. This is highlighted by an up-regulation of IL-10 mRNA and protein levels in IFNAR1^−/−^ mice compared with WT mice after TBI. IL-10 is an anti-inflammatory cytokine, and is produced by immune cells undergoing a phenotypic switch from “resting” to “alternatively activated”. Consequently, IL-10 is used as an M2 marker (Wang et al., 2013). These alternately activated cells exhibit anti-inflammatory, proangiogenic features, and up-regulate various different cytokines and growth factors (Sica et al., 2006). In a mid cerebral artery model of stroke, IL-10 administration was found to suppress overexpression of proinflammatory mediators IFNγ and TNFα and reduce lesion volume (Liesz et al., 2009). Furthermore, IL-10 administration in a mouse model of TBI improved neurological recovery post-TBI, and was associated with reduced levels of TNFα and IL-1β expression in the cortex and hippocampus (Knoblach and Faden, 1998). These studies demonstrate that an increased anti-inflammatory environment via increased IL-10 can result in a shift toward suppression of a proinflammatory environment and consequent protection and may explain how increased IL-10 levels in the IFNAR1^−/−^ after TBI contributes to the resultant neuroprotection.

Additional to silencing type-1 IFN signaling genetically, we demonstrated protective effects of administering a blocking monoclonal antibody, MAR1. MAR1-treated WT mice demonstrated reduced infarct volumes, similar to IFNAR1^−/−^ mice. Furthermore, administration of MAR1 resulted in decreased production of the proinflammatory cytokines IL-1β and IL-6. Secondary production of the type-1 IFNs, IFNα, and β, was also blocked by MAR1 administration. Finally, MAR1 treatment improved behavioral outcome in WT mice after TBI. Collectively, these results suggest that MAR1 treatment is effective at managing TBI-induced neuroinflammation, leading to a beneficial post-traumatic outcome, and indicates its potential as a viable therapeutic for combating TBI. There is little doubt that in our model of TBI which has a disrupted blood–brain barrier that the MAR-1 antibody is able to cross readily in to the brain. Whether this will occur with an intact barrier is unknown at this time. It is known that some monoclonal antibodies are able to enter the brain (Yu et al., 2011). What we do know from our bone marrow chimera experiments is that the hematopoietic compartment influences TBI outcome and IFNAR1 signaling is heavily implicated. This data suggests that it is possible that MAR1 does not need to cross the barrier, and its peripheral effects on type-1 IFN signaling contribute to neuroprotection in TBI. Future studies are underway to address this interesting mechanistic question.

To further investigate the cellular mechanisms leading to protection following TBI in the IFNAR1^−/−^ mice, bone marrow chimeras were generated. Through the development of bone marrow chimeras, we ascertained that removal of type-1 IFN signaling in hematopoietic cells conferred protection after TBI. This finding proposes that hematopoietic cells are a large contributor to the deleterious aspects of type-1 IFN signaling after TBI. Many studies have used chimeric approaches to investigate the relative contribution of brain-derived and peripheral cells in injury responses (Chen et al., 2012; Downes et al., 2013). These studies have implicated various inflammatory pathways, in both resident and peripheral immune cells after injury. The distinction between these two tissue compartments is an important factor to consider when trialing therapeutics for acute brain injuries. Furthermore, targeting novel neuroprotectants to cell types outside of the CNS may extend the window of therapeutic opportunity in the treatment of TBI.

We have clearly demonstrated a critical role for type-1 IFN signaling in TBI. Importantly, removal of type-1 IFN signaling, both genetically and pharmacologically confers protection after TBI. Detailed investigation into this response after TBI indicates that signaling in peripheral immune cells is crucial in driving the deleterious neuroinflammatory cascade. Therefore, it is evident that specific targeting of the peripheral component of the type-1 IFN response represents a promising therapeutic option after brain injury.

## Synthesis

The decision was a result of the Reviewing Editor Quentin Pittman and the peer reviewers coming together and discussing their recommendations until a consensus was reached. A fact-based synthesis statement explaining their decision and outlining what is needed to prepare a revision is listed below. The following reviewers agreed to reveal their identity: V. Wee Yong, Clive Svendsen

The research supports a belief that reducing the inflammatory cascade after TBI will lead to reduced mortality. Although the author's central thesis is not novel and the attempt to reduce a specific cytokine by knockout and moAb injections has been attempted with a number of cytokines, this manuscript does describe a detrimental role for type 1 IFN signaling in TBI. The reviewers indicate that the experiments are generally well done, with adequate sample size mostly, and statistical analysis is appropriate. The reviewers advise me that the following revisions would improve the manuscript:

(1) Please discuss whether you believe that MAR1 crossed the blood brain barrier; it might after TBI, and perhaps you could stain for it. Alternatively, the reported positive changes noted with MAR1 Ab could be due to systemic inhibition, and it is important to clarify whether the moAb acts centrally or systemically.

(2) The abstract and methods say 8-week old mice were used. This age in mice is quite young for TBI models. The mean weight noted, 23 grams, is about 11 weeks, which is still too young and too small. Perhaps the authors could expand on why the used such young, small mice.

(3) "Improved neurological function" is based on a 5 second analysis of gait post-injury. Please provide evidence either from the literature or your own experiments that this assessment of motor function provides data similar to the more familiar rotorod or open field test results.

(4) In Figure 2, the lesion should be traced out to help readers evaluate how the authors describe a given area as an infarcted zone. More clarity should also be provided by the authors as to what constitutes the parameters that enabled them to call a given area an infarcted zone.

(5) Methods for quantifying GFAP and Iba1 do not specify how many sections were assessed and in what regions of the infarct. It is possible that different areas of the infarct are assessed. A description of section selection is necessary. While intensity analysis might be informative in terms of reactive GFAP+ and Iba1+ cells, the method of using intensity analysis for quantification of histological markers is not traditional and a more quantitative approach (unbiased stereology) would be desirable to determine total numbers of inflammatory cells. Please comment and/or justify your approach. Furthermore, for the quantitation of GFAP and IBA1 staining, the intensity of fluorescence should be normalized to an internal control in order to control for variability across different sections/slides/day of staining.

(6) In Figure 1a, where it is mentioned that there is an upregulation of IFNb at 24 hours after TBI in wild type mice, that statement should be re-worded since the "upregulation" is only when comparing between wild type and knockout mice. When compared to SHAM, there is no upregulation of IFNb at 24h after TBI.

(7) Although the graph in figure 9 shows the data, there is no comment in the results or discussion section regarding lack of significant change in infarct volume of IFNAR1 -/- mice reconstituted with WT bone marrow.

(8) Are there any data on the number of dead neurons (e.g. by fluoro-jade B staining) since volume could be reflective of edema, astrocyte hypertrophy, rather than death of neurons.?

(9) That there is increase in IBA1 immuno-reactivity in knockout mice is interesting and is counter-intuitive, as there is less injury. Hints of an M2 upregulation is provided by the cytokine increase of IL-10. It would be important for the authors to stain for an M2 marker in their study (e.g. arginase-1, CD204, CD206).

(10) In Figure 7a where the administration of the function blocking antibody MAR1 resulted in decreased IFN levels and a dampened pro-inflammatory response, are there any data on IL-10? Did that go up?

Minor:

(1) There was no disclosure statement, which begs the question as to whether the MAR1 moAb were supplied through a relationship with Leinco. Does anybody in the lab have ties to the company?

(2) For discussion, a mouse after a single unilateral injury invariable recovers fully, are the authors proposing that the injury was permanent and the KO or moAb allowed for cure (unlikely) or are the authors proposing that the KO/moAb led to a quicker recovery (more likely)?
